# Non-Conveyance Due to Patient-Initiated Refusal in Emergency Medical Services: A Retrospective Population-Based Registry Analysis Study in Riyadh Province, Saudi Arabia

**DOI:** 10.3390/ijerph18179252

**Published:** 2021-09-02

**Authors:** Hassan N. Moafa, Sander M. J. van Kuijk, Mohammed E. Moukhyer, Dhafer M. Alqahtani, Harm R. Haak

**Affiliations:** 1 Department of Health Services Management, Faculty of Public Health and Tropical Medicine, Jazan University, Jazan 82817 2820, Saudi Arabia; 2 Department of Health Services Research, CAPHRI School for Public Health and Primary Care, Maastricht University, 6229 GT Maastricht, The Netherlands; h.haak@mmc.nl; 3 Department of Clinical Epidemiology and Medical Technology Assessment, Maastricht University Medical Centre, 6202 AZ Maastricht, The Netherlands; sander.van.kuijk@mumc.nl; 4 Department of Academic Development and Quality, Faculty of Applied Medical Sciences, Jazan University, Jazan 82511, Saudi Arabia; mmoukhyer@jazanu.edu.sa; 5 Department of Electronic Transaction Management, Saudi Red Crescent Authority, Ministry of Health, Riyadh 13251-8261, Saudi Arabia; mdhafer@srca.org.sa; 6 Department of Internal Medicine, Maxima Medisch Centre, 5631 BM Eindhoven, The Netherlands; 7 Department of Internal Medicine, Maastricht University Medical Centre, 6229 HX Maastricht, The Netherlands

**Keywords:** emergency medical services, non-conveyance, patient-initiated refusal, emergencies, Riyadh, Saudi Arabia

## Abstract

This research study aimed to investigate the association between demographic and operational factors and emergency medical services (EMS) missions ending in non-conveyance (NC) due to patient-initiated refusal (PIR). We conducted a retrospective population-based registry study by analyzing 67,620 EMS missions dispatched to the scene during 2018 in the Riyadh province. First, the number and percentages of conveyances statuses were calculated. Then, using crude and adjusted linear and logistic regression analysis, we determined which characteristics were predictors of NC due to PIR. We found that 23,991 (34.4%) of missions ended in NC due to PIR, and 5969 ended in EMS-initiated refusal (8.6%). NC rates due to PIR were higher for women, adults, for missions in Riyadh city, during nighttime, for medical emergencies, and for advanced life support (ALS) crews. We also found the following additional predictors significantly associated with the odds of NC due to PIR in crude regression analyses: age category, geographical location, EMS-shift, time of call, emergency type, and response time. We conclude that the NC rate represents half of all missions for patients requesting EMS, and the rate in Riyadh city has increased compared to previous studies. Most NC cases occur for the highest urgency level of medical emergency type in Riyadh city during the nighttime with ALS crews. NC due to PIR involves younger patients more than elderly, and females more than males. This study’s findings have provided empirical evidence that indicate that conducting further studies involving EMS providers, patients, and the public to identify precise and detailed reasons is required.

## 1. Introduction

Emergency medical services (EMS) crews can decide not to convey non-emergency patients to emergency departments (EDs) and only treat them at the scene, even if they had been triaged by phone as being high-acuity [1] This non-conveyance (NC) by EMS-initiated refusal (EIR) reduces the burden on EDs and increase the benefits for the patient by avoiding the possibility of getting infections during transportation and in the ED [2,3,4,5]. Disadvantages of NC due to EIR are evident when potentially subsequent events occur for the patient which jeopardizes their safety, especially during the first 48 h after initiating refusal [6,7]. Many EMS providers do not give clear guidance supporting decisions at the scene [8,9,10,11]. Therefore, many paramedics avoid legal accountability that may arise due to a possible wrong or risky decision of non-conveying patients [3,4,12].

On the other hand, patients may refuse to be transported by EMS to EDs, against clinical advice, for different reasons. This is called NC due to patient-initiated refusal (PIR) [13,14]. Reasons for PIR may be related to different factors such as a previous negative experience, concern over ED waiting time, or just wanting medical reassurance for their health status [15]. Besides, the unaffordability of the chargeable cost of ambulance transportation and treatment in the EDs can be another reason for refusal [16]. Family refusal may also be a factor due to their conviction that going to the EDs is unnecessary, or because they prefer using another transportation method [17]. Like unnecessary transportation of non-emergency patients, patients’ or their relatives’ refusal of transportation against medical advice is considered a waste of resources, and it delays the response for other patients on the dispatching waiting list, and subsequently patients may expose themselves to unnecessary risk of serious complications.

NC rates have gradually increased worldwide during the last two decades. Between 2002 and 2016, it increased from 33% to 40–68% in the different geographical regions in the UK [5,18]. However, those studies may not be generalizable to the Saudi population because the Saudi EMS system is still under development. Saudi patients can refuse transportation by ambulance against medical advice, and EMS providers have to accept patients’ decisions accordingly without referring to assigned online medical control at the dispatch center. The situation in the US is different, where approving the patient’s refusal requires communicating with online medical management [14]. Too often, people in Saudi Arabia (SA) travel to an ED without medical referral using their own transportation. Consequently, Saudi hospitals’ EDs suffer from overcrowding as 53% of patients are non-emergencies [19]. The Saudi EMS system is overburdened with a high volume of 30.9% of low acuity emergency patients transported to EDs [20].

A study in 2014 found that the NC rates due to PIR in Riyadh city of SA was over 50% [21]. However, a more recent study from 2017 showed that the rate had increased to 59.9%, but found no association between sex, age and geographical area of the patient, and the likelihood of NC [22]. Nevertheless, none of those studies investigated the relationship of operational factors nor the urgency levels of missions or services-level type provided as factors that may have associations with the NC events.

The present study aimed to assess the distribution of conveyance status of solo EMS missions that were individually dispatched by ground ambulances in Saudi Arabia and to investigate the association of demographic and operational factors of missions to those being ended with NC status due to PIR.

## 2. Materials and Methods

### 2.1. Study Design and Setting

This retrospective population-based registry study was conducted in the Riyadh province of SA from 1 January 2018, to 31 December 2018. All EMS database records in the Saudi Red Crescent Computer-aided Dispatching (SRCCAD) system (developed by Sahab Company, Riyadh, Saudi Arabia) for completed missions were used for the analysis.

The Saudi Red Crescent Authority (SRCA) provides emergency services through a double-tier EMS system around the clock and free of charge for all people without considering sex, religion, ethnicity, or socioeconomic status. Riyadh’s EMS responds to all different calls, including non-emergency cases, through a call and dispatch center using a free-of-charge number. The dispatchers in the call center use a computer-aided dispatch (CAD) system to support decision-making and nominate the appropriate crew. Riyadh’s EMS provides medical aid in two service-levels: basic life support (BLS) and advanced life support (ALS). BLS and ALS are available in the capital city of Riyadh, while the other small cities and rural areas only have BLS. BLS ambulances are crewed with two emergency medical technicians. The ALS crew is equipped with one paramedic, with two years of additional training, and with a medical doctor.

The study proposal was reviewed and approved by the ethical committee at Jazan University with the registry number: REC39/9-S084. The Ethics Committee concluded that informed consent was not required because of the anonymity of the data collected for routine ambulance missions and the study’s retrospective design. Privacy and confidentiality were considered during manipulating data of the study.

### 2.2. Data Collection

The EMS database includes patients’ demographic characteristics, geographical locations, types of emergency, type of dispatched crews, and the outcomes of missions that ended with either NC or transportation to hospitals’ EDs. It also includes all consecutive time intervals starting from patients’ call to the call center and ending by the departure of EMS crews for hospitals’ EDs. Call-takers manage all data entry in the call center by collecting information from patients or their relatives and EMS crews after they arrived at the scene. In addition, EMS crews provide data related to time intervals to the call center as part of monitoring time management.

The data were obtained through the operations and information department in the Riyadh EMS branch of the SRCA. Data were exported to an encrypted Microsoft Excel file and converted to an IBM SPSS file (version 25.0 Armonk, NY: IBM Corp) for further analyses.

### 2.3. Selection of Participants

All EMS missions dispatched to the scene for different types of emergencies that occurred between 1 January 2018, and 31 December 2018, in the Riyadh province in SA were included. We excluded EMS missions for incidents to which more than one crew was dispatched, planned EMS missions (e.g., for large gatherings), and missions that had missing data on patient sex, age, geographical location, or hospital. We defined non-conveyance status as those EMS missions where EMS providers contact patients, followed by the decision made by patients, EMS providers, or both that EMS missions for those patients would be ended at the scene without ambulance transportation. Non-conveyance due to PIR are patients who requested EMS and refused EMS transportation against medical advice. Non-conveyance due to EIR are those patients who call for EMS, and EMS providers decided that the patients’ conditions were not an emergency or can be treated at the scene, and hence patients agreed to those decisions. Conversely, conveyed cases were defined as those patients who requested the EMS and were transported by the ambulances to hospitals’ EDs according to medical advice. According to those definitions, we excluded EMS missions ended by NC due to other reasons that lead to NC, in which the EMS providers had arrived at the scene, and patients were not contacted verbally, due to one of the following reasons: hoax call, other EMS caregivers had transported the patient, the patient was not found at the scene, the patient had been conveyed by air ambulance, or when the reason was not documented. Ultimately, we included the three conveyance statuses; conveyed cases, NC due to PIR, and NC due to EIR for the primary analysis. For subsequent analysis, we selected only those that received advice to be transported, i.e., we omitted those missions that were ended by non-conveyance due to EIR.

### 2.4. Methods of Measurement

The SRCCAD system saves data and creates different time variables like response time, on-scene time, time from activation of EMS to departure from the scene, travel time to the hospital, the time of patient handover to emergency departments, and total EMS time. In addition, the system saves demographic information such as sex, age, and incident location based on information provided by emergency attendants or patients themselves. The CAD prioritizes missions into three levels: highly urgent, intermediate, and non-urgent, and dispatches crews accordingly. We included available demographic information besides specific periods related to response time, on-scene time, and the activation of the EMS to depart to the scene time (the total response and on-scene time together). Age was categorized into three groups (<15 years as children, 15–<60 years as adult, ≥60 years for elderly) according to Saudi Statistical Authority’s definition of age categories [23]. We categorized geographical locations based on the last Saudi Statistical Authority report published in 2010, into urban (>5000 inhabitants) and rural (≤5000 inhabitants) [23]. We categorized the urban areas into two sub-classifications: Riyadh city (5.2 millions) and other smaller cities (>5000–<500,000 inhabitants). We considered the association of outcomes for EMS missions, whether they ended by conveyance or non-conveyance statuses, with the time factors related to the daily operational periods by EMS providers or the time when patients request EMS support. Therefore, we added other essential variables related to the EMS daily shift and the specific periods when patients request EMS support. We then categorized the daily EMS shifts into two periods: daytime shift and nighttime shift. The daytime shift starts from 09:00 a.m. to 8:59 p.m., and the nighttime shift starts from 9:00 p.m. to 08:59 a.m. the next day. Additionally, to identify the influence of weekends compared to weekdays, we categorized days of the week into working days (Sunday to Thursday in SA) and the weekends (Friday and Saturday). Finally, we stratified the calling times of the patients for EMS support into two categories office time and rest time. Office time is the patients’ callings time for EMS from Sunday to Thursday at 8:00 a.m. to 4:00 p.m., and rest time is patients calling times for EMS from Sunday to Thursday at 4:01 p.m. to 7:59 a.m. the next day, in addition to the weekends. We have clustered emergencies built-in CAD into five categories; medical emergencies, trauma emergencies, psychiatric emergencies, gynecological emergencies, and non-emergencies. Category of crews involved in ambulance service-level, whether BLS or ALS crews, were included. The three urgency levels produced by CAD were included as high priority for high-acuity, intermediate priority for intermediate-acuity, and low priority for low-acuity cases.

### 2.5. Primary Data Analysis

A table with column-wise percentages for NC due to PIR was calculated and presented as Supplementary Table S1. We calculated differences in number and percentages of subgroups of the characteristics of the mission and the patient between PIR, EIR and conveyed cases. We calculated them by rows adding up to 100% in order to assess the difference in the distribution of different categories of variables (e.g., males and females) over the different conveyed statuses. Additionally, we tested the difference using Pearson’s chi-square test. The Mann–Whitney U Test was used to compare response time and on-scene time, and total mission time between PIR and conveyed cases.

Next, we selected only those missions in which the patient was advised by the EMS personnel to be transported to the hospital (i.e., EIR missions were omitted). We conducted logistic regression to assess the association between variables and the odds of PIR in comparison to transported cases, assuming those combined represent all patients for whom transportation was warranted. Missions’ outcomes were treated as binary variables (conveyed cases as the reference). Unadjusted odds ratios (ORs) and adjusted ORs (AORs) with 95% confidence interval (CI) were calculated. After univariable, or unadjusted, logistic regression, all characteristics were added to the model to assess the adjusted association between characteristics and PIR. We considered *p <* 0.05 as statistically significant for all analyses.

## 3. Results

### 3.1. Characteristics of Study Subjects

**Figure 1:**
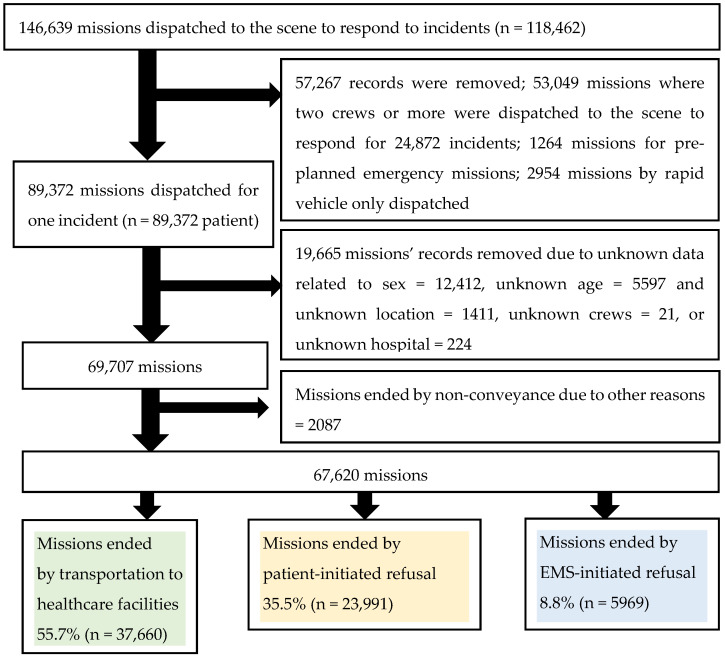
Flow chart of included and excluded missions that ended with the three outcomes.

Of the 67,620 included patients, 37,660 (55.7%) were transported to a healthcare facility, and the remainder were not conveyed either due to PIR or EIR. Of the complete sample, NC due to PIR was recorded for 35.5%, while NC due to EIR was recorded for 8.8% of patients. Of all NC missions, 80.1% was due to PIR, and 19.9% due to EIR (Figure 1).

Of total solo EMS records (n = 67,620), 64% of missions accounted for males, 36% females, 4% children, 62% adult, 34% elderly, 82.8% from Riyadh capital city, 13.2% small cities, and 4% from rural areas.

### 3.2. Main Results

Table 1 shows characteristics of missions and patients stratified by conveyance status. Note that rows add up to 100%, so that the distribution of different categories of variables (e.g., males and females) over the different conveyed statuses can be assessed. All results of chi-square tests were significant, indicating associations between the variables on the left and the categories of conveyance (conveyed, PIR, and EIR). Of all male patients who requested EMS (n = 43,258), 55.2% were conveyed to EDs of hospitals, 35.5% ended by NC due to PIR, and 9.5% ended by NC due to EIR. Of female patients (n = 24,362) who requested EMS support, 56.8% were conveyed to hospitals’ EDs, 35.5% ended by NC due to PIR, and 7.7% ended by NC due to EIR. Of total EMS missions for children (n = 2714, median age = 8 years), 51.1% were conveyed to hospitals’ EDs, 38.6% ended by NC due to PIR, and 10.2% ended by NC due to EIR. Of total EMS missions for adults (n = 41,908, median age 33 years), 51.1% were conveyed, 38.7 ended by NC due to PIR, and 10.2% ended by NC due to EIR. Of total EMS missions for the elderly (n = 22,998, median age = 70 years), 64.6% were conveyed to EDs, 29.3% ended by NC due to PIR, and 6.1% ended by EIR. The most pronounced differences in PIR rates for different categories of characteristics were found for age categories, geographical locations, patient emergency type, and EMS crew type. We found that PIR rates were significantly lower in the elderly (29.3%), during nighttime shifts (36.6%), for gynecological emergencies (13.9%), and when dispatching BLS crews (34.6%). For EIR, differences in rates were significantly lower for females, the elderly, office times, gynecological emergencies, and for lower priority missions. A table with column-wise percentages can be found in Supplementary Table S1.

The median response time for NC cases due to patient refusal was 18.8 min (IQR; 13.8–26.0 min), which was slightly longer than the median for the conveyed patients (18.3 min, IQR; 13.1–25.5), *p*-value *<* 0.001. The median on-scene period was longer for non-conveyed patients due to PIR (22.2 min IQR; 15.4–31.8), in comparison to conveyed patients (19.3 min, IQR; 12.0–27.5 min), *p*-value *<* 0.001 (Table 2).

Table 3 shows the findings of the crude and adjusted logistic regression models for the demographic and operational predictors; age category, geographical location, daytime EMS-shift, time of call, emergency type, ambulance crew type, urgency level, and response time were all significantly associated with the odds of NC due to PIR in the crude regression analyses. After adjusting the model, we found that age category, geographical location, EMS shift, daily work, emergency type, and response time remained significantly associated with PIR. EMS missions for elderly patients were less likely to end by NC due to PIR in comparison to EMS missions for adults (OR: 0.60; 95% CI 0.58 to 0.62), (AOR: 0.53; 95% CI 0.51 to 0.55). Compared to missions dispatched in the capital city of Riyadh, we found that EMS missions in other smaller cities and urban locations were less likely to end by NC due to PIR in both crude and adjusted models’ analysis. When compared to non-emergency cases, missions for gynecological emergencies were less likely to end by NC due to PIR (OR: 0.28; 95% CI 0.22 to 0.34), (AOR: 0.14 95% CI 0.06 to 0.30). Furthermore, EMS missions during the nighttime shift were more likely to end by NC due to PIR (OR: 1.11; 95% CI 1.08 to 1.15) (AOR: 1.05; 95% CI 1.01 to 1.09).

## 4. Discussion

This study’s main goal was to investigate the association of demographic and operational factors of EMS missions and the likelihood of those missions being ended by NC due to PIR in the Riyadh province of SA. Our study showed that the NC rate due to PIR was more frequent for EMS-missions dispatched to females, children and adults (compared to the elderly), in Riyadh city, during the nighttime shift, with medical emergencies, ALS crews, high priority missions, and longer response times. The NC rate was 44.3% in comparison to 55.7% conveyed patients. It is worth noting that when we combine NC due to PIR and EIR together, the rate of NC in our study can be placed in the middle range of the very broad variation rate of 3.7% to 93.7%, provided in the systematic review of Ebben et al. [11]. Nevertheless, given that 8.8% of NC was due to EIR in our data (n = 5969), compared to developed countries the rate can be considered relatively low. However, if the non-emergency patients (n = 15,232) had been treated and discharged at the scene instead of being transported to the hospital, the EIR rate would have been 31%. The Saudi EMS system is loaded by the burden of transporting more than half of non-emergency cases to hospitals’ EDs [20]. Therefore, reorganizing the EMS system by developing specific training programs for EMS providers and providing them with operational support would empower their compliance to implement NC protocols for non-emergency cases [10]. Consequently, a future implementation might reduce unnecessary transportation and increase the NC rate due to EIR, and improve patients’ health. Moreover, it would reduce the burden on Saudi hospitals’ emergency departments. Calling for EMS support and refusing transportation against medical advice afterward is deemed an inappropriate use of EMS because it deprives EMS providers of rest, food, and education and delays the response time for other cases [24,25]. Kawakami et al. found that a lack of knowledge of EMS is considered one of the socioeconomic factors that leads to significant unnecessary ambulance calls [26]. Community education activities about the appropriate use of ambulance services and finding alternative means for non-emergency cases were effective in reducing unnecessary EMS calls, reducing operational costs, and improving response time [27,28]. A Saudi study revealed that 33% of Saudi people are unaware of the free-of-charge emergency telephone number (997) [29,30]. To the best of our knowledge, public awareness in SA about when to call EMS was not evaluated. Therefore, educating people about when to call EMS and the potential complications of refusing medical aid are still necessary in SA.

Research comparing NC between urban and rural areas is limited, and most studies investigated NC in an urban setting [11]. Our study reveals that the NC rate in urban locations (44.7%) was higher than in rural areas (33.8%). This is in line with a Dutch study that reported NC rates in urban areas to be 57.3%, much higher compared to rural areas (26.1%), and was concluded as well in a systematic review of studies from different regions of the world from the same group [11,31]. Similarly, a Canadian study revealed that 29.5% of total NC cases accounted for rural areas and 47.9% for urban areas, while 22.5% were not indicated [32].

We found that the rate of NC due to PIR was predominant (80.1%) compared to EIR (19.9%). This prevalence of NC due to PIR was found at a higher rate in the capital city of Riyadh (80.9%), compared to small cities (73.8%) and rural areas (73.7%). Our finding greatly exceeds a previous estimate of NC due to PIR of 54.1% found in Riyadh city by Alrazeeni et al. in 2014 [21]. On the other hand, another small sample study in 2017 by Alanazy et al. found even higher rates compared to ours: 89.7% in Riyadh city and 78.2% in smaller cities and rural locations compared to other reasons of NC [22]. Our explanation for this difference in rates may be the small sample size and unintentional selection bias in their study [22].

Our study’s crude and adjusted logistic models demonstrated that EMS missions for patients in small cities and rural areas were associated with lower odds of ending missions by NC due to PIR compared to EMS missions in Riyadh city. Therefore, besides previous studies, our study indicates that a phenomenon of NC due to PIR needs to be addressed by conducting further research involving the public and patients [33].

EMS missions that ended with NC due to PIR were significantly more often for female patients than for males, while EMS missions that ended with NC due to EIR were significantly more for male patients than for females. In the crude and adjusted regression models, EMS missions for females were not significantly associated with the odds of NC due to PIR compared to males. Our study results are supported by R. Waldron et al., who found that patient sex was not related to NC due to PIR [34].

Of all NC due to PIR, we found that PIR accounted for 28.1% for the elderly, 67.5% for adults, and 4.4% for children. This finding is in line with a US study by Holder et al., which found that NC due to PIR accounted for 25% of elderly patients and 75% for children and adults (less than 60 years old) [35]. We found that 64.6% of EMS missions for elderly patients were conveyed to hospitals’ EDs, 29.3% had NC due to PIR status, and 6.1% had NC due to EIR status. However, of all NC records for the elderly, 17.2% were recorded due to EIR and 82.8% due to PIR. Our study is in line with a US study conducted by Vilke et al., who found that 19% of the NC rate for the elderly was due to EIR, while 77% were due to PIR [16]. The logistic model for both crude data and after adjustment showed that EMS missions for elderly patients were significantly less likely to be associated with the odds of NC due to PIR. This significant association between elderly patients and the low rate of NC due to PIR is consistent with several studies showing the high association between elderly and the transportation by ambulances to EDs [36,37,38,39,40]. Oosterwold J et al., in their systematic review, found that the severity of the medical condition for elderly patients was not the main predictor of conveyance, but there were different factors related to EMS structure and related to the patients themselves [41]. However, 70% of the elderly who refused transportation would still need medical follow-up, are more likely to request EMS again within 72 h, and might die within seven days of their refusal [16]. Therefore, we recommend further research to investigate elderly expectations about EMS and their satisfaction.

Children represent 4% of this study’s setting. Compared to the 51.1% conveyed children, we found EMS missions ended by NC due to PIR (parents’ refusal) accounts for 38.6%, and 10.6% for NC due to EIR. Of all NC records for children, 79% of children’s NC rate was due to parents’ refusal and 21% due to EIR. Parents’ refusal was also found in other countries. For example, a Canadian study showed that 54% of the NC rate for children was due to parent refusal and 29.2% due to EIR [17]. Our study showed that EMS missions for children were not associated with the odds of NC due to PIR. Further studies are needed to identify factors related to parents’ refusal to transport their children.

Research related to NC due to PIR of obstetrics and gynecological emergencies is scarce [42,43,44]. We found that 13.9% of EMS records for gynecological emergencies showed NC due to PIR; the reasons for which are unclear, but may be related to the gender of EMS providers as 100% of crews in Saudi EMS are staffed all-male [45]. A US study revealed that regardless of patients’ gender, NC due to PIR rates for all-male EMT crews were much higher (odds ratio of 4.75) compared to all-female and mixed-gender EMT crews [34]. Perhaps in SA, this difference is much higher, especially since most Saudi females prefer female physicians to treat them for internal medicine, gynecological disease, and emergencies [46,47]. However, no evidence of Saudi females patients’ preference regarding gender type of EMS providers was found. Moreover, the database for this study was limited to providing information related to refusal due to the gender of EMS providers. Therefore, we think further studies involving women are still needed.

Our study showed that the EMS missions that ended with NC due to PIR were relatively more frequent during the nighttime shift. EMS missions during office time were found to be less likely associated with NC due to PIR. Snooks et al. found that the NC rate decreased during the last hour of the EMS shift [48]; however, our study found that the last hour of the daytime shift did not show a drop in NC rate due to PIR. On the contrary, we found that EMS missions ending with NC due to PIR affectedly increased between 17:00 and 23:00, including 20:00 to 21:00 at the end of the daytime shift.

The response time was significantly longer for non-conveyed patients than conveyed patients. Both the crude and adjusted models’ analyses showed that the increase in response time was associated with NC due to PIR. Short response time is an extreme priority for high-acuity cases. High-acuity cases in our study showed that there was no association with NC due to PIR.

Our study has several limitations; the retrospective design being one. The database was designed for operational purposes, and most of the information related to the crew’s timeline information was documented in an automated way via wireless communication between dispatchers and EMS crews at the scene. However, in the case of network failure, data related to patients’ locations and timeline are missed. Combined with human underperformance of on-time documentation in the call center, we had to exclude 22% of missions. Our exclusion for those missing data might have induced unintended selection bias. Similarly, we excluded the NC for other reasons because they were not related to our definition of NC and cannot be accounted for any one of both types of NC (NC due to PIR and NC due to EIR). Thus, our exclusion of the NC due to other reasons (2.9%) means that our results may only be generalized to PIR and EIR, and not to other types of NC. For example, we cannot categorize those patients transported by other providers as NC due to PIR or NC due to EIR because, basically, those patients were not contacted or treated at the site by EMS ground ambulance providers. Nevertheless, using the database has provided our study with good statistical power to identify between-group differences and the associations of the available demographic and operational characteristics with the conveyance outcomes of patients. The study lacks detailed sub-classifications of PIR that were not available in the database, such as explaining the patients’ views regarding their refusal. In addition, for some records, operational circumstances or demographics were missing, perhaps due to working load or network failure, leading to the exclusion of records. In addition, since the SRCA is a member of the International Federation of Red Cross and Red Crescent Societies, questioning about race and socioeconomic status is prohibited and was therefore not available [49]. These characteristics may be associated with conveyance status. Another limitation was that the EMS database could not be linked to other Riyadh hospitals and primary care centers. Therefore, we were not able to identify the patient’s health consequences after they refused transportation.

## 5. Conclusions

This is the first Saudi study that analyzed the EMS registry to investigate NC due to PIR for every patient who requested ground ambulance services in the Riyadh province of SA. In addition, this study showed that half of all emergency patients requesting EMS ended up not being conveyed to the hospital. Most NC cases occurred for the highest urgency level of medical emergency types in Riyadh city during the nighttime with ALS crews. NC due to PIR involved male more than female patients, adult patients more than elderly and children, and Riyadh city more than other areas. However, relatively (percentage-wise), EMS missions for the gender category of female patients, the age category of elderly, and the geography of small cities were predominant to end up with conveyance status. Most NC due to PIR occurred during the daytime shift, and also more occurred during rest time compared to office time. Medical emergencies had the highest NC due to PIR. The rate of NC due to PIR has increased in Riyadh capital city compared to previous studies. This study’s findings have provided empirical evidence that might indicate that conducting further studies involving EMS providers, patients, and the public to identify precise, detailed reasons is required. We recommend linking the EMS database for those patients who refused transportation against medical advice with their registries located in other health sectors due to the necessity of identifying the medical consequences as they occur.

## Figures and Tables

**Table 1:** Characteristic feature of demographic, operational factors for 67,620 missions dispatched at the scene.

Variable	Number N = 67,620	Conveyed Cases N = 37,660 (55.7%) &	Patient-Initiated Refusal for Non-Conveyance N= 23,991 (35.5%) &	EMS-Initiated Refusal for Non-Conveyance N = 5969 (8.8%) &
Sex				
Male	43,258	23,823 (55.1)	15,346 (35.5)	4089 (9.5)
Female	24,362	13,837 (56.8)	8645 (35.5)	1880 (7.7)
**Age category**				
Child ≤ 15 y	2714	1388 (51.1)	1048 (38.6)	278 (10.2)
Adult	41,908	21,410 (51.1)	16,205 (38.7)	4293 (10.2)
Elderly ≥ 60 y	22,998	14,862 (64.6)	6738 (29.3)	1398 (6.1)
**Geographical location**				
Riyadh city	55,991	29,494 (52.7)	21,437 (38.3)	5060 (9.0)
Small cities	8899	6360 (71.5)	1873 (21.0)	666 (7.5)
Rural ≤ 5000	2730	1806 (66.2)	681 (24.9)	243 (8.9)
**EMS Shift**				
Daytime	37,544	21,350 (56.9)	12,982 (34.6)	3212 (8.6)
Nighttime	30,076	16,310 (54.2)	11,009 (36.6)	2757 (9.2)
**Week**				
Weekday	48,618	27,178 (55.9)	17,264 (35.5)	4176 (8.6)
Weekend	19,002	10,482 (55.2)	6727 (35.4%)	1793 (9.4)
**Time of call**				
Rest time	50,104	27,559 (55.0)	18,008 (35.9)	4537 (9.1)
Office time	17,516	10,101 (57.7)	5983 (34.2)	1432 (8.2)
**Emergency type**				
Non-emergencies	26,398	15,232 (57.7)	9227 (35.0)	1939 (7.3)
Medical	25,817	13,241 (51.3)	10,111(39.2)	2465 (9.5)
Trauma	14,040	8252 (58.8)	4324 (30.8)	1464 (10.4)
Psychiatric	697	378 (54.2)	236 (33.9)	83 (11.9)
Gynecological	668	557 (83.4)	93 (13.9)	18 (2.7)
**Crew type**				
BLS	50,190	28,499 (56.8)	17,365 (34.6)	4326 (8.6)
ALS	17,430	9161 (52.6)	6626 (38.0)	1643 (9.4)
**Urgency level**				
High-priority	29,447	15,893 (54.0)	10,680 (36.3)	2874 (9.8)
Intermediate	11,744	6516 (55.5)	4074 (34.7)	1154 (9.8)
Low-priority	26,429	15,251 (57.7)	9237 (35.0)	1941 (7.3)

ALS, Advance Life support; BLS, Basic Life Support. ^&^ All variables show significance difference at *p*-value level < 0.05 between variables and conveyance status.

**Table 2:** Timeline differences between transported and non-conveyance emergencies due to patient-initiated refusal (n = 61,651).

Time Line	Conveyed Cases 37,660	Non-Conveyed Cases 23,991	*p*-Value
Response time median (IQR)	18.3 (13.1–25.5)	18.8 (13.8–26.0)	*<* 0.001
On-scene time median (IQR)	19.3 (12.0–27.5)	22.2 (15.4–31.8)	*<* 0.001
Total time ^†^ median (IQR)	39.4 (29.3–51.1)	43.2 (33.4–55.8)	*<* 0.001

^†^ The total time from patients activated the emergency call number until the crew leaves the scene. IQR, Interquartile Range.

**Table 3:** Crude and adjusted associations between characteristics of the patient or mission, and patient-initiated refusal (n = 61,651).

Variable	N(%) of NC due to PIR, N = 23,991	Crude		Adjusted *	
		OR (95% CI)	*p*-Value	OR (95% CI)	*p*-Value
**Sex**					
*Male (ref)*	15,346 (64.0)				
Female	8645 (36.0)	0.97 (0.94–1.00)	0.075	1.01 (0.99–1.07)	0.115
**Age**					
*Adult (ref)*	1048 (4.4)				
Child < 15 y	16,205 (67.5)	1.00 (0.92–1.08)	0.954	0.96 (0.90–1.06)	0.560
Elderly ≥ 60 y	6738 (28.1)	0.60 (0.58–0.62)	*<* 0.001	0.53 (0.51–0.55)	<0.001
**Location**					
*Riyadh city (ref)*	21,437 (89.4)				
Small cities < 500K	1873 (7.8)	0.41 (0.38–0.43)	<0.001	0.40 (0.38–0.43)	<0.001
Rural ≤ 5000 people	681 (2.8)	0.52 (0.47–0.57)	<0.001	0.50 (0.45–0.55)	<0.001
**EMS Shift**					
*Daytime (ref)*	12,982 (54.1)				
Nighttime	11,009 (45.9)	1.11 (1.08–1.15)	<0.001	1.05 (1.01–1.09)	0.007
**Week**					
*Weekday (ref)*	17,264 (72.0)				
Weekend	6727 (28)	1.01 (0.98–1.05)	0.577	0.99 (0.95–1.03)	0.538
**Time of call**					
*Rest time (ref)*	18,008 (75.1)				
Office time	5983 (24.9)	0.91 (0.87–0.94)	<0.001	0.94 (0.90–0.99)	0.009
**Emergency type**					
*Non-emergencies (ref)*	9227 (38.5)				
Medical	10,111 (42.1)	1.26 (1.21–1.31)	<0.001	0.83 (0.38–1.81)	0.632
Trauma	4324 (18.0)	0.87 (0.83–0.91)	<0.001	0.48 (0.22–1.05)	0.065
Psychiatric	236 (1.0)	1.03 (0.88–1.22)	0.719	0.48 (0.22–1.07)	0.074
Gynecological	93 (0.4)	0.28 (0.22–0.34)	<0.001	0.14 (0.06–0.30)	<0.001
**Crews type**					
*BLS (ref)*	17,365 (72.4)				
ALS	6626 (27.6)	1.19 (1.14–1.23)	<0.001	0.97 (0.93–1.01)	0.104
**Urgency level**					
*High priority (ref)*	10,680 (44.5)				
Intermediate	4074 (17.0)	0.93 (0.89–0.97)	0.002	0.99 (0.94–1.04)	0.706
Low priority	9237 (38.5)	0.90 (0.87–0.93)	<0.001	0.65 (0.30–1.42)	0.282
**EMS Time**					
Response time		1.00 (1.00–1.01)	<0.001	1.00 (1.00–1.01)	<0.001

* Adjusted for all variables in the crude model. ^&^ conveyed patients (n = 37,660) as the reference. ALS, Advance Life support; BLS, Basic Life Support; NC, non-conveyance; PIR, patient-initiated refusal.

## Data Availability

Data are available on request from the corresponding author upon reasonable request. The data are not publicly available due to privacy.

## Supplementary Materials

The following are available online at https://www.mdpi.com/article/10.3390/ijerph18179252/s1, Table S1. Distribution of non-conveyance due to patient-intieated refusal (n = 23,991).
