# Supplementary material for: Non-Conveyance Due to Patient-Initiated Refusal in Emergency Medical Services: A Retrospective Population-Based Registry Analysis Study in Riyadh Province, Saudi Arabia
Source: Int J Environ Res Public Health. 2021 Sep 2;18(17):9252. doi: 10.3390/ijerph18179252 (PMC8431479; doi:10.3390/ijerph18179252)
Supplement: Supplementary file 1 [file ijerph-18-09252-s001.zip › ijerph-1334045-supplementary.pdf]

Supplementary Table 1 presents the detailed information of the distribution of EMS records for NC due to PIR (n= 23991) stratified by different categories of characteristic variables related to patients and missions. Therefore, of total EMS missions ended by NC due to PIR (n=23991), 64% were males, while females were 36%. Children accounted for 4.4%, adults 67.5%, and elderly 28.1%. Urbancitiy of patients' locations showed that 89.4% of all NC due to PIR were from Riyadh's capital city, 7.8% from small cities, and 2.8% from rural areas. In addition, 54.1% of all NC due to PIR accounted for daytime shifts, whereas 45.9% were during nighttime shifts. 72.4% of all NC due to PIR were dispatched by BLS crews, while 44.5% were dispatched as high urgent of priority.

Table S1. Distribution of non-conveyance due to patient-intieated refusal (n= 23,991)

Characteristics	Number	Percentages
**Sex**		
Male	15346	64.0 %
Female	8645	36.0 %
**Age category**		
Child < 15 y	1048	4.4 %
Adult ≥ 15 - < 60 y	16205	67.5 %
Elderly ≥ 60 y	6738	28.1 %
**Patients location**		
Riyadh city 5	21437	89.4 %
Small cities	1873	7.8 %
Rural ⩽ 5000	681	2.8 %
**EMS Shift**		
Daytime	12982	54.1 %
Nighttime	11009	45.9 %
**Week**		
Weekday	17264	72.0 %
Weekend	6727	28.0 %
**Works period**		
Rest time	18008	75.1 %
Office time	5983	24.9 %
**Emergency types**		
Non-emergencies	9227	38.5 %
Medical	10111	42.1 %
Trauma	4324	18.0 %
Psychiatric	236	1.0 %
Gynecological	93	0.4 %
**Crews type**		
Basic life support	17365	72.4 %
Advanced life suuport	6626	27.6 %
**Urgency levels**		
High- priority	10680	44.5 %
Intermediate	4074	17.0 %
Low-priority	9237	38.5 %
